# Composition of Fatty Acids and Localization of *SREBP1* and *ELOVL2* Genes in Cauda Epididymides of Hu Sheep with Different Fertility

**DOI:** 10.3390/ani12233302

**Published:** 2022-11-26

**Authors:** Jiamei Liu, Wanhong Li, Xiuxiu Weng, Xiangpeng Yue, Fadi Li

**Affiliations:** 1 State Key Laboratory of Herbage Improvement and Grassland Agro-Ecosystems, Key Laboratory of Grassland Livestock Industry Innovation, Ministry of Agriculture and Rural Affairs, Engineering Research Center of Grassland Industry, Ministry of Education, College of Pastoral Agriculture Science and Technology, Lanzhou University, Lanzhou 730020, China; 2 Gansu Runmu Biological Engineering Co., Ltd., Jinchang 737200, China

**Keywords:** Hu sheep, epididymis, fertility, crude fat, fatty acid

## Abstract

**Simple Summary:**

The Chinese Hu sheep is well-known for its high fertility and early sexual maturity. Rams reach sexual maturity at the age of six months. This study investigated differential crude fat content, fatty acid composition and related metabolism genes localization and expression in cauda epididymis between high and low fertility Hu sheep at six months old. The crude fat content was positively correlated with epididymal weight. High expression of *SREBP1* and *EVOVL2* in high fertility Hu sheep may lead to the higher n-3 PUFA content, especially DHA, providing a basis for understanding the relationship between fertility and sperm storage environment.

**Abstract:**

The epididymis is an organ that transports, matures and stores sperm, and has functions such as secretion and absorption. Polyunsaturated fatty acid (PUFA) compositions in sperm membrane were changed during the process of epididymis maturation and influence the male fertility. This study aimed to investigate differences in crude fat and fatty acid content in cauda epididymis between high and low fertility of Hu sheep. One hundred and seventy-nine Hu ram lambs were fed from 56 days to 6 months under the same environment. After the feeding trial, all rams were slaughtered, and the body weight, testicular weight, epididymal weight and sperm density were measured. Pearson correlation analysis showed significantly moderate positive correlation between epididymal weight and sperm density and testicular weight. Eighteen rams were selected and divided into the high fertility group (H, *n* = 9) and low fertility group (L, *n* = 9) according to the epididymal weight, sperm density and histomorphology. The crude fat content, fatty acid profile and genes related to fatty acid metabolism were detected. The crude fat content, total fatty acid, total n-3 PUFA and docosahexaenoic acid (C22:6n-3, DHA) content of cauda epididymis in high fertility group was significantly higher than those in low fertility group (*p* < 0.05). However, the ratio of n-6/n-3 PUFA was significantly lower than that in group L (*p* < 0.05). Immunohistochemistry results showed that SREBP1 and ELOVL2 were expressed in pseudostratified columnar ciliated epithelium and smooth muscle cells. The mRNA expression of *SREBP1* (*p* = 0.09) and *ELOVL2* (*p* < 0.05) in the high fertility group were increased. In conclusion, the high expression of *SREBP1* and *ELOVL2* may contribute to high n-3 PUFA content in cauda epididymis of high-fertility Hu sheep.

## 1. Introduction

Lipid metabolism is critical to physiological and cellular processes in the male reproductive system, including sperm motility activation, capacitation and acrosome reaction [1]. The lipid content of epididymosomes in cauda epididymis changed with the increase of sphingomyelin and the decrease of cholesterol, compared to caput epididymis [2]. The sperm in the caput epididymis has a high concentration of saturated fatty acid, while the unsaturated fatty acid is dominant in the sperm in the cauda epididymis [3]. Docosahexaenoic acid (C22:6n-3, DHA) accounts for 50% of the total content of polyunsaturated fatty acids (PUFAs) in sperm [4].

The predominance of unsaturated fatty acids in the sperm is closely related to the mobility of the sperm membrane and the ability to acquire forward motion [5], which improves the fertilization ability of sperm [6]. n-3 PUFA, especially DHA, has a positive correlation with sperm fertilization ability [6]. Studies have shown that PUFAs can be considered as markers of sperm fertility and pathology [7]. In addition, PUFA is the precursor of prostaglandin synthesis and can regulate the expression of key enzymes involved in steroid metabolism [8]. The appropriate concentration of prostaglandin can maintain the normal morphology and function of sperm [9] and increase the sperm number by enhancing the movement of sperm from epididymis to vas deferens, which is conducive to ejaculation [10].

The long chain PUFA in mammal cell membrane phospholipids is derived from diet taking linoleic acid (C18:2n-6, LA) and α- linolenic acid (C18:3n-3, ALA) as the substrate. Under the catalysis of fatty acid desaturase 1 and 2 (FADS1, FADS2) and elongation of very long chain fatty acids 2 and 5 (ELOVL2, ELOVL5), ALA generates n-3 PUFAs such as eicosapentaenoic acid (C20:5n-3) and DHA, while LA uses the same enzyme system to synthesize n-6 PUFAs such as arachidonic acid (C20:4n-6, AA) and docosapentaenoic acid (C22:5n-6, DPA) [11]. As the place of sperm storage, there are rare studies on the fatty acid content and composition on the cauda epididymis of sheep.

For further investigation, the correlation among the body weight, testicular weight, epididymal weight, sperm density and crude fat content from one hundred and seventy-nine Hu ram lambs aged six months old, were analyzed. After that, eighteen rams were selected and divided into high and low fertility according to epididymal weight, sperm density and histomorphology as we described previously [12], and fatty acid content was detected by gas chromatograph. Immunohistochemistry and qRT-PCR were carried out to detect the expression of fatty acid synthesis genes, such as *SREBP1* and *ELOVL2* in cauda epididymis.

## 2. Materials and Methods

### 2.1. Reagents

Methyl heneicosanoate (Code No. 51535-1G) was purchased from Sigma-Aldrich (St. Louis, MO, USA). Thirty-seven FAME standards were purchased from Supelco (Bellefonte, PA, USA). OBCFA standards (BR3 and ME 93) were purchased from Larodan Fine Chemicals (Malmö, Sweden). Goat serum (Code No. WGAR1009-5) and DAB chromogenic kit (Code No. G1212) were purchased from Servicebio (Wuhan, China). Rabbit anti-SREBP1 polyclonal antibody (Code No. bs-1402R) and rabbit anti-ELOVL2 polyclonal antibody (Code No. bs-7053R) were purchased from Bioss (Beijing, China). TransZol Up (Code No. ET111-01), 2× PerfectStart^TM^ Green qPCR SuperMix (Code No. AQ601) were purchased from TransGen Biotech (Beijing, China). PrimeScript^TM^ RT reagent kit with a gDNA Eraser (Perfect Real Time) kit (Code No. RR047A) was purchased from Takara (Dalian, China). Primers were synthesized by Augct Biotechnology (Beijing, China).

### 2.2. Animals and Sample Collection

One hundred and seventy-nine healthy Hu ram lambs were selected and immunized under a standardized immunization program before they were weaned at fifty-six days. All rams were reared indoors and housed in individual pens (0.8 × 1 m) at Minqin Defu Agriculture Co. Ltd. (Minqin, China) to six months. They were fed with the same commercial pellet feed and water ad libitum, and then slaughtered as previously described [12]. Testes and epididymides were stripped of white membranes and separated, rinsed well with saline and dried with gauze. After that, the testes and epididymides were weighed bilaterally and the long and short axes of the testes were measured. A portion of the left cauda epididymal tissue was preserved in liquid nitrogen for molecular experiments and a portion was stored in a sealing bag at −20 °C for lipid analysis experiments. And another part was fixed in formaldehyde. Sperm in the cauda epididymis were collected and counted as previously described [13].

A total of eighteen individuals were selected and divided into the low fertility group (L, *n* = 9) and high fertility group (H, *n* = 9) according to epididymal weight, sperm density and histomorphology, as previously described [12].

### 2.3. Determination of Crude Fat Content and Fatty Acid Profiles

Crude fat content in cauda epididymis of one hundred twenty rams was measured with the Soxhlet method as previously described [14]. Fatty acid content in the freeze-dried epididymal tissue (0.2 g) were extracted using chloroform/methanol extraction methods referred to O’Fallon et al. [15]. Methyl heneicosanoate (C21:0) was used as internal standard adding to hexane to the final concentration of 1 mg/mL. Fatty acids methyl esters (FAME) were quantified by gas chromatograph (Thermo Scientific, Milan, Italy), which was equipped with a flame ionization detector and a 100 m × 0.25 mm × 0.20 μm fused silica capillary column (HP-88; Agilent Technologies, Co., Ltd., Santa Clara, CA, USA). The temperature was held at 50 °C for 4 min, increased to 175 °C at 13 °C/min, held for 27 min, and finally increased at 3 °C/min to 215 °C. The determination and analysis of fatty acids in samples were based on the 37 FAME standards and OBCFA standards.

### 2.4. Immunohistochemistry

Formaldehyde-fixed cauda epididymal tissue from three sheep in each group were dehydrated, clear, and paraffin-embedded, then cut into 4 μm tissue sections, dewaxed and placed in citrate buffer (pH 6.0) in a microwave oven. The sections were added 3% hydrogen peroxide solution, closed with rabbit serum and incubated overnight at 4 °C in primary antibody SREBP1 (1:200), ELOVL2 (1:200) diluted with PBS, while the negative control was incubated with PBS only. Then sections were incubated with horseradish peroxidase (HRP)-labeled secondary antibody, added DAB chromogenic solution, stained with hematoxylin, divided with hematoxylin differentiation solution, immersed in hematoxylin blue-return solution, and then dehydrated, transparent, and finally sealed with neutral resin. Images were observed under microscope (Olympus, Tokyo, Japan), acquired at a magnification of 400×, and then calculated by using Image Pro Plus. The average optical density (AOD) was calculated as follows: AOD = integrated optic density/area.

### 2.5. RNA Extraction and cDNA Synthesis

Total RNA was extracted from cauda epididymis of nine sheep in each group by using TransZol Up. RNA samples met the criterion as OD260/280 within 1.8–2.0 and OD260/230 higher than 2.0 were used for following experiments. Reverse transcription reaction was performed according to the instructions of PrimeScript^TM^ RT reagent kit with a gDNA Eraser (Perfect Real Time) kit. The reaction mixtures contained 1 μg of total RNA, 1 μL of gDNA eraser, 2 μL of 5× gDNA eraser buffer, and 6 μL RNase-free water. The mixture was incubated at 42 °C for 2 min, and then added with 1 μL of PrimeScript RT enzyme mix, 1 μL of RT primer mix, 4 μL of PrimeScript buffer, and 4 μL of RNase-free water. The mixture was incubated at 42 °C for 25 min followed by 85 °C for 5 s. DNase-free water was used to dilute cDNA for 20 times. cDNA was stored at 80 °C until use.

### 2.6. Quantitative Real-Time PCR Analysis

The reaction mixtures contained 1 μL of cDNA (10 ng/μL), 1 μL of primers (Table 1), 10 μL of 2× PerfectStart^TM^ Green qPCR SuperMix and 8 μL of DNase-free water. The reaction steps were as follows: initial denaturation at 95 °C for 5 min, followed by 40 cycles at 95 °C for 5 s and 61 °C for 30 s. Melting curve analysis was used to verify the amplification of a single product. Reference genes, *hypoxanthine phosphoribosyltransferase 1* (*HPRT1*), *ribosomal protein S18* (*RPS18*) and *ribosomal protein lateral stalk subunit P2* (*RPLP2*) were used to normalize the expression level. The relative expression analysis of *SREBP1* and *ELOVL2* was calculated using the 2^−^^ΔΔCt^ method.

### 2.7. Statistical Analyses

The experimental results were presented as mean ± standard error of mean. Data were evaluated using IBM SPSS 22.0 (SPSS, Chicago, IL, USA). Pearson correlation analysis was performed by GraphPad Prism 8.2.1 (GraphPad, CA, USA). Significant differences were analyzed by independent samples *t*-test. *p* value < 0.05 was statistically significant.

## 3. Results

### 3.1. Correlation Analysis of Body Weight, Sperm Density, Testicular and Epididymal Weight in Hu Sheep

**Figure 1:**
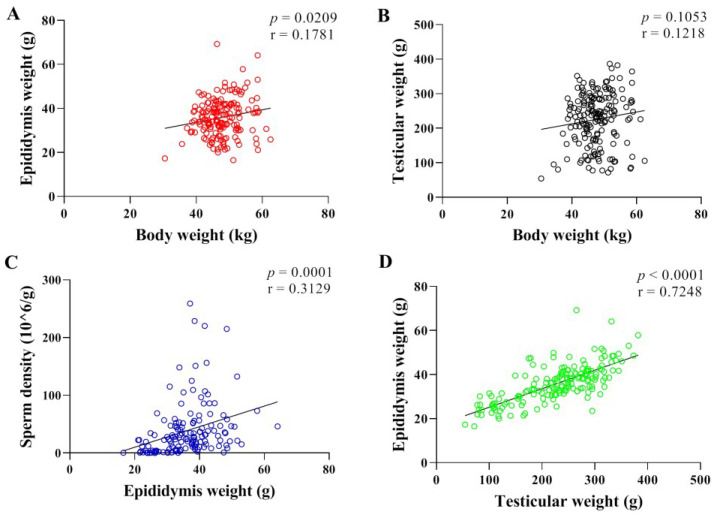
Correlation analysis between reproductive traits. (**A**): Body weight–epididymal weight (*n* = 168). (**B**): Body weight–testicular weight (*n* = 178). (**C**): Epididymal weight–sperm density (*n* = 145). (**D**): Testicular weight–epididymal weight (*n* = 168).

Due to the damage caused to the sample during the slaughter process, some data including one cryptorchidism were eliminated. As shown in Table 2, the average weight of six-month-old Hu sheep was 47.69 kg (30.50–62.50 kg). The average left testicular weight was 113.30 g (29.00–193.90 g) and the average left epididymal weight was 18.03 g (9.00–39.00 g). The correlation analysis of body weight, testicular weight, epididymal weight and sperm density showed that there was a significant weak positive correlation between body weight and epididymal weight (Figure 1A, *p* = 0.0209, r = 0.1781), and there was no significant correlation between body weight and testicular weight (Figure 1B, *p* = 0.1053, r = 0.1218). There was extremely significantly moderate positive correlation between epididymal weight and sperm density (Figure 1C, *p* = 0.0001, r = 0.3129) and the correlation between testicular weight and epididymal weight was extremely significantly strongly positive (Figure 1D, *p* < 0.0001, r = 0.7248).

Eighteen individuals from the high and low fertility groups as we studied previously were selected for further analysis. The left testicular weight (162.79 ± 3.06 vs. 56.09 ± 5.45 g), epididymal weight (23.76 ± 1.97 vs. 11.87 ± 0.78 g) and sperm concentration (65.99 ± 27.27 × 10^6^/g vs.3.44 ± 2.40 × 10^6^/g) in the H group was significantly higher respectively than that in the L group (*p* < 0.01). The diameter of epididymal ducts in the H group was significantly larger than that in the L group (0.62 ± 0.02 mm vs. 0.45 ± 0.01 mm, *p* < 0.01) [12].

### 3.2. Crude Fat Content

**Figure 2:**
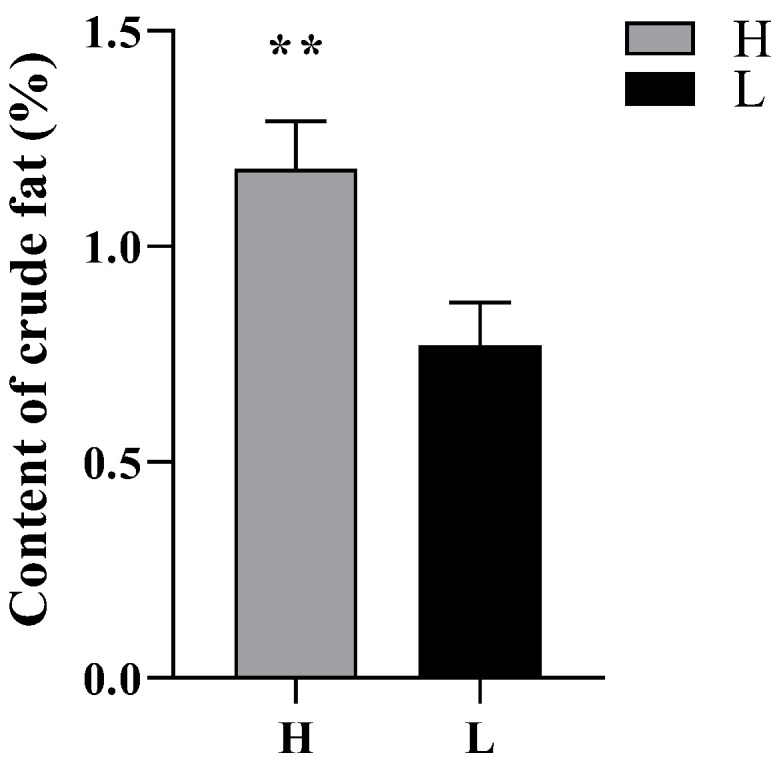
Crude fat content of cauda epididymis of Hu sheep with different fertility. ** indicates *p* < 0.01.

Crude fat content showed a significant positive correlation with sperm density (r = 0.28, *p* < 0.01) and epididymal weight (r = 0.20, *p* < 0.05). The crude fat content of group H was extremely significantly higher than that of group L (*p* < 0.01, Figure 2).

### 3.3. Fatty Acid Content

The cauda epididymis of group H contained about 20 mg/g saturated fatty acids (SFA), 3 mg/g monounsaturated fatty acids (MUFA), nearly 11 mg/g PUFA, 8 mg/g n-6 PUFA, and 2 mg/g n-3 PUFA, and the total fatty acid content was higher in group H than that in group L (*p* = 0.032). In terms of fatty acid composition, C16:0 (*p* = 0.059) and C22:0 (*p* = 0.050) were higher in group H than those in group L. The C22:6n-3, total n-3 PUFA content was significantly higher (*p* < 0.001) and the n-6/n-3 PUFA ratio in group H was significantly lower (*p* = 0.014) than that in group L (Table 3).

### 3.4. Immunohistochemistry

**Figure 3:**
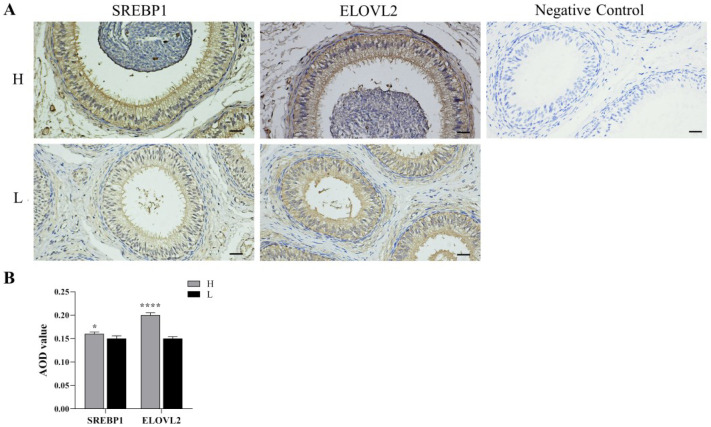
Distribution of SREBP1 and ELOVL2 in cauda epididymis of Hu sheep with different fertility. (**A**): Localization of immunopositive signal (400×), scale bar = 20 μm. (**B**): AOD value of immunopositive signal. * indicates *p* < 0.05, **** indicates *p* < 0.0001.

SREBP1 and ELOVL2 proteins were expressed in the pseudostratified columnar ciliated epithelium of the epididymal ducts as well as in the smooth muscle cells around the ducts, while only a small amount of expression was observed in the connective tissue between the epididymal ducts. The AOD values of SREBP1 and ELOVL2 in the pseudostratified columnar ciliated epithelium were significantly higher in group H than those in group L (*p* < 0.05, Figure 3).

### 3.5. Relative Expression of SREBP1and ELOVL2 Genes

**Figure 4:**
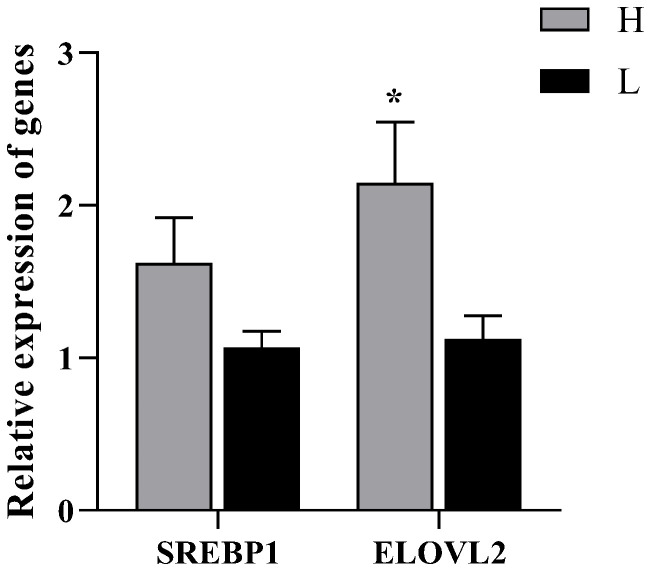
mRNA relative expression of *SREBP1* and *ELOVL2* in cauda epididymis of Hu sheep with different fertility. * indicates *p* < 0.05.

*ELOVL2* expression was significantly up-regulated at the mRNA level in the group H (*p* = 0.02), and also *SREBP1* expression was higher in the group H (*p* = 0.09, Figure 4).

## 4. Discussion

In the present study, the correlation analysis between epididymal weight and sperm density was extremely significantly moderate positive and the correlation between testicular weight and epididymal weight was extremely significantly strong positive. Our previously published article showed testicular weight, epididymal weight, sperm concentration and histological observation of high and low fertility groups, while the data presented that high fertility rams were accompanied by larger testes and epididymides [12]. Since the Sertoli cells in the testis proliferate rapidly during the prepubertal period [16], and the number of spermatozoa that can be attached to each Sertoli cell is certain, the number of Sertoli cells determines the spermatogenesis capacity and the size of the testis. Studies have shown that individuals with larger testes have higher sperm viability. In cattle, testis size was positively associated with sperm production, percentage of normal sperm, and fertility rate of cows [17]. These studies suggest that testis size can be used as a basis for assessing sperm production and quality, and can be used as a criterion for judging the fertility of breeding rams. Rams with high fertility produce more spermatozoa, which are transported through the epididymal duct to reach the cauda epididymis for storage, hence the larger diameter of the epididymal duct, the higher number of spermatozoa in the cauda epididymis and the heavier weight of the epididymis. Studies on rhesus monkeys has shown that fertility can be judged by epididymal weight and epididymal sperm count [18].

We observed that the lambs were fed in the same diets while the crude fat content of the cauda epididymis in group H was significantly higher than that in group L. It has been recorded that the balance of lipid synthesis in epididymal epithelium during sperm maturation can maintain sperm lipid homeostasis [5], and PUFAs can modify sperm-membrane fatty acids and regulate sperm-membrane structure [19,20]. In the present study, the high content of DHA and total n-3 PUFA within group H might be beneficial to the extension of sperm membrane, thus enhancing sperm viability. In practical production, the addition of n-3 PUFAs to the diets of male animals such as pigs, cattle and buffaloes increased the glutathione content and superoxide dismutase activity of the organism, increased antioxidant capacity and improved semen quality [21,22]. There is a competitive relationship between n-3 and n-6 PUFAs metabolism in mammals. The high content of n-6 PUFAs will lead to the loss of n-3 PUFAs, resulting in the imbalance of the proportion of essential fatty acids and their derivatives. The results of our study showed that the n-6/n-3 PUFA ratio was significantly lower in group H than that in group L, indicating that the content and distribution of n-3 PUFA in cauda epididymis in group H was superior, which might be beneficial to enhance the sperm viability [23].

SREBP1 is a key transcription regulator of genes related to lipid metabolism [24], coordinating the composition of membrane lipids and controlling the synthesis of unsaturated fatty acids and cholesterol. The overexpression of *SREBP1* gene promotes the synthesis and accumulation of fatty acids and triglycerides in goat mammary epithelial cells and elevates the levels of C16:0 and C18:1 [25], while it is negatively regulated by MUFA and PUFA. Incubating HEK-293 cells with unsaturated fatty acids can suppress the expression of *SREBP1* [26]. Dietary n-3 PUFA inhibits adipogenesis by accelerating phosphorylation of nSREBP1 through 26S proteasome and Erk-dependent pathways [27]. In the present study, *SREBP1* was up-regulated at the mRNA level in group H compared to group L, which could facilitate the production and accumulation of PUFA.

ELOVL2 protein catalyzes the synthesis of C22:5n-3 from C20:5n-3. Spermatogenesis arrest occurs in *ELOVL2* knockout mice, and serum n-3 and n-6 of C20 and C22 PUFA levels were abnormal [28]. Overexpression of *ELOVL2* in cultured primary hepatocytes enhances C20:5n-3 to C24:5n-3 conversion, thereby attenuating C20:5n-3-mediated PPARα-regulated transcription and increasing the nuclear content of *SREBP1c* [29]. In the present study, *ELOVL2* in group H was significantly higher than that in group L at mRNA level, and the protein level and AOD values also showed an up-regulation trend, indicating active synthesis of long chain PUFA. ELOVL2 expressed in the pseudostratified columnar ciliated epithelium as well as in the connective tissue, similar to the study on rabbits [30], and the number of marked epididymal vesicles was higher in rabbits fed flaxseed diet. Dietary treatment of rams with linseed oil increased the expression levels of *SREBP1* and *ELOVL2*, stimulated the estradiol secretion, and thus improved the testis development [31].

## 5. Conclusions

The crude fat content was positively correlated with the epididymal weight of six-month-old Hu lambs. The cauda epididymis of the high fertility group was rich in n-3 PUFA, and the n-6/n-3 PUFA ratio was significantly lower than that of the low fertility group. The expression of *SREBP1* and *ELOVL2* was up-regulated in the high fertility group, which was conducive to the synthesis of n-3 PUFA, providing a theoretical basis for the selection and breeding of high fertility rams.

## Figures and Tables

**Table 1:** Primer sequences used for qRT-PCR.

Gene (ID)	Primer Sequence (5′-3′)	Product Size (bp)
*HPRT1*	F: CGACTGGCTCGAGATGTGAT	197
XM_015105023.2	R: TCACCTGTTGACTGGTCGTT	
*RPS18*	F: CACTGAGGACGAGGTGGAAC	186
XM_004018745.3	R: CTGTGGGCCCGAATCTTCTT	
*RPLP2*	F: AGCGCCAAGGACATCAAAAAG	136
XM_004023349.4	R: TGGCCAGCTTGCCGATAC	
*SREBP1*	F: ATGGCTTTGATTCTCGTGGC	202
XM_012151742.2	R: TTTTCAGTGTCCGCAACTGG	
*ELOVL2*	F: ACAGACCTGCTCTTTCCCTC	114
XM_015093202.1	R: TGTAGCCTCCTTCCCAACTG	

ELOVL2 = elongation of very long chain fatty acids-like 2; HPRT1 = hypoxanthine phosphoribosyl transferase 1; RPLP2 = ribosomal protein lateral stalk subunit P2; RPS18 = ribosomal protein S18; SREBP1 = sterol regulatory element binding protein 1.

**Table 2:** Descriptive statistics of reproductive traits of six-month-old Hu sheep.

Traits	Mean	95% Confidence Interval for Mean		Min	Max	SD	CV	Number
		Lower	Upper					
Body weight (kg)	47.69	46.89	48.49	30.50	62.50	5.42	0.11	178
Left testicular weight (g)	113.30	107.62	118.98	29.00	193.90	38.41	0.34	178
Right testicular weight (g)	112.70	107.05	118.35	25.60	193.70	38.22	0.34	178
Left testicular index (g/kg)	2.39	2.27	2.51	0.70	4.00	0.82	0.34	178
Right testicular index (g/kg)	2.38	2.26	2.50	0.68	4.47	0.81	0.34	178
Left epididymal weight (g)	18.03	17.33	18.73	9.00	39.00	4.65	0.26	171
Right epididymal weight (g)	17.72	17.07	18.38	6.30	30.10	4.36	0.25	174
Left epididymal index (g/kg)	0.38	0.37	0.39	0.17	0.84	0.10	0.25	171
Right epididymal index (g/kg)	0.37	0.36	0.39	0.12	0.65	0.09	0.24	174
Sperm density (10^6^/g)	38.26	30.65	45.87	0.06	259.19	46.69	1.22	147

Testicular index (g/kg) = testicular weight/body weight; epididymal index (g/kg) = epididymal weight/body weight.

**Table 3:** Fatty acid profiles in cauda epididymis of Hu sheep with different fertility.

Fatty Acid Content (mg/g)	H	L	*p*-Value
C14:0	0.813 ± 0.078	0.632 ± 0.057	0.078
C15:0	0.050 ± 0.004	0.048 ± 0.003	0.731
C16:0	4.851 ± 0.229	4.315 ± 0.131	0.059
C16:1	0.094 ± 0.005	0.091 ± 0.007	0.740
C17:0	0.148 ± 0.008	0.131 ± 0.005	0.099
C18:0	3.747 ± 0.153	3.693 ± 0.134	0.795
C18:1n9t	0.032 ± 0.002	0.050 ± 0.008	0.060
C18:1n9c	2.747 ± 0.124	2.709 ± 0.109	0.822
C18:2n6t	0.005 ± 0.001	0.005 ± 0.000	0.331
C18:2n6c	1.376 ± 0.088	1.366 ± 0.055	0.923
C20:0	0.124 ± 0.007	0.133 ± 0.006	0.330
C18:3n6	0.019 ± 0.002	0.025 ± 0.003	0.074
C20:1	0.114 ± 0.007	0.110 ± 0.006	0.707
C18:3n3	0.031 ± 0.002	0.031 ± 0.001	0.776
C20:2	0.170 ± 0.030	0.159 ± 0.012	0.750
C22:0	0.057 ± 0.002	0.064 ± 0.003	0.050
C20:3n6	0.756 ± 0.057	0.630 ± 0.031	0.073
C20:3n3	0.004 ± 0.000	0.003 ± 0.000	0.089
C20:4n6	6.534 ± 0.185	5.797 ± 1.096	0.526
C23:0	0.107 ± 0.005	0.103 ± 0.003	0.513
C22:2	0.006 ± 0.001	0.005 ± 0.000	0.827
C24:0	0.043 ± 0.004	0.054 ± 0.007	0.216
C20:5n3	0.044 ± 0.026	0.030 ± 0.011	0.607
C24:1	0.013 ± 0.001	0.014 ± 0.001	0.132
C22:6n3	2.812 ± 0.215	1.275 ± 0.165	<0.001
Total	34.607 ± 0.727	31.388 ± 1.157	0.032
SFA	19.852 ± 0.444	19.087 ± 0.286	0.167
MUFA	3.000 ± 0.131	2.975 ± 0.120	0.892
PUFA	11.755 ± 0.398	9.325 ± 1.144	0.073
n-3 PUFA	2.891 ± 0.217	1.338 ± 0.165	<0.001
n-6 PUFA	8.689 ± 0.270	7.823 ± 1.080	0.457
n-6/n-3 PUFA	3.148 ± 0.252	6.158 ± 0.956	0.014

## Data Availability

Not applicable.
